# Obesity-related drug transporter expression alterations in human liver and kidneys

**DOI:** 10.1007/s43440-024-00665-7

**Published:** 2024-10-16

**Authors:** Katarzyna Kosicka-Noworzyń, Aleksandra Romaniuk-Drapała, Yi-Hua Sheng, Christine Yohn, Luigi Brunetti, Leonid Kagan

**Affiliations:** 1 Department of Physical Pharmacy and Pharmacokinetics, Poznan University of Medical Sciences, Rokietnicka 3, Poznań, 60-806 Poland; 2 Department of Clinical Chemistry and Molecular Diagnostics, Poznan University of Medical Sciences, Rokietnicka 3, Poznań, 60-806 Poland; 3 Department of Pharmaceutics, Ernest Mario School of Pharmacy, Rutgers, The State University of New Jersey, 160 Frelinghuysen Road, Piscataway, NJ 08854 USA; 4 Department of Pharmacy Practice and Administration, Ernest Mario School of Pharmacy, Rutgers, The State University of New Jersey, 160 Frelinghuysen Road, Piscataway, NJ 08854 USA; 5 Center of Excellence for Pharmaceutical Translational Research and Education, Ernest Mario School of Pharmacy, Rutgers, The State University of New Jersey, 160 Frelinghuysen Road, Piscataway, NJ 08854 USA

**Keywords:** Drug transporters, Pharmacokinetics, Obesity, Renal clearance, Hepatic clearance

## Abstract

**Background:**

Pathophysiological changes associated with obesity might impact various drug pharmacokinetics (PK) parameters. The liver and kidneys are the primary organs involved in drug clearance, and the function of hepatic and renal transporters is critical to efficient drug elimination (or reabsorption). Considering the impact of an increased BMI on the drug’s PK is crucial in directing dosing decisions. Given the critical role of transporters in drug biodisposition, this study investigated how overweight and obesity affect the gene expression of renal and hepatic drug transporters.

**Methods:**

Human liver and kidney samples were collected post-mortem from 32 to 28 individuals, respectively, which were divided into the control group (lean subjects; 18.5 ≤ BMI < 25 kg/m^2^) and the study group (overweight/obese subjects; BMI ≥ 25 kg/m^2^). Real-time quantitative PCR was performed for the analysis of 84 drug transporters.

**Results:**

Our results show significant changes in the expression of genes involved in human transporters, both renal and hepatic. In liver tissue, we found that *ABCC4* was up-regulated in overweight/obese subjects. In kidney tissue, up-regulation was only observed for *ABCC10*, while the other differentially expressed genes were down-regulated: *ABCA1*, *ABCC3*, and *SLC15A1*.

**Conclusions:**

The observed alterations may be reflected by the differences in drug PK between lean and obese populations. However, these findings need further evaluation through the proteomic and functional study of these transporters in this patient population.

**Supplementary Information:**

The online version contains supplementary material available at 10.1007/s43440-024-00665-7.

## Introduction

Currently, about 42% of the US population is obese (body mass index [BMI] > 30 kg/m^2^), while more than 9% have been reported to be morbidly obese (BMI > 40 kg/m^2^). Obesity-related conditions, including heart disease, stroke, type 2 diabetes, and certain types of cancer, rank among the leading causes of preventable, premature death [1]. Predictions of the World Obesity Atlas suggest that by 2035, over 4 billion people worldwide, constituting more than 51% of the global population, will have BMI ≥ 25 kg/m² and be considered overweight or obese [2]. Multiple comorbidities are common in obesity and often lead to polypharmacy [3]. As a consequence, potential drug interactions may be present in up to 50% of individuals with obesity [4].

Patients with obesity experience several pathophysiological changes that may influence the pharmacokinetics (PK) of drugs. The pharmacokinetic process consists of four main steps; the first is drug absorption, which influences the initial amount of drug in the body. Altered body composition and increased adiposity can lead to changes in gastric emptying and intestinal transit times, affecting the rate and extent of drug absorption. The second step is drug distribution, mainly characterized by the volume of distribution (V_d_). This element is probably the most impacted by obesity-related changes [5]. Additionally, obese patients have relatively less lean and more fat tissue per kilogram of total body weight than non-obese individuals. Lower lean body mass relative to overall body weight potentially causes a significant increase in the V_d_ for lipophilic drugs and a low or moderate increase of this parameter for hydrophilic drugs. Altered PK of gentamicin and vancomycin in obese patients were linked with significantly impacted V_d_ [6–8]. Moreover, nitrazepam elimination half-life increased significantly due to an increase in V_d_ in obese patients compared with lean subjects [9]. The third phase of PK includes all biotransformation reactions, leading to increased hydrophilicity and elimination. The last step is the excretion, which is primarily urinary and fecal, and more rarely cutaneous, pulmonary, and by other secretions. The last two phases, metabolism and excretion, are characterized by the same pharmacokinetic parameter, the elimination clearance (CL). The drug CL depends on metabolic clearance, particularly in the liver and/or renal clearance through the kidney (CL_h_ or CL_r_, respectively) [5]. Renal elimination is the main route of drug excretion and involves all the nephron processes: glomerular filtration, tubular secretion, and tubular reabsorption. This stage may also be influenced by glomerular hyperfiltration, which has been reported in obese individuals [10, 11]. The altered tubular secretion and reabsorption were listed as causes of increased excretion of ciprofloxacin and cimetidine in obese individuals [12].

The mass of some organs, such as the lung and thyroid, do not vary with the gain of body weight, while others, such as the liver, heart, kidney, and spleen, significantly increase with the increasing BMI [13]. Furthermore, increased cardiac output and liver blood flow substantially impact the net effect of obesity on drug metabolism [10, 14]. The liver is a primary organ for xenobiotic metabolic degradation, involving multiple processes. Hepatic drug transporters, belonging to the ATP-binding cassette (ABC) or solute carrier (SLC) transporter families, are significant elements of drug hepatobiliary elimination [15, 16]. These transporters facilitate drug uptake from the blood at the sinusoidal membrane of hepatocytes and their secretion into bile at the canalicular membrane [17]. Additionally, certain drugs or their metabolites are transported by sinusoidal ABC transporters back into the blood for renal elimination [18]. The kidney, another critical pharmacokinetic organ, encompasses metabolism, excretion, and transport of small molecules, drugs, and xenobiotics [19]. In the kidney, ABCs (efflux transporters) and SLCs (most of them are uptake transporters) recognize and transport metabolites, hormones, and organic substrates, including pharmaceutical agents. Some members of these subfamilies significantly influence a drug’s disposition, efficacy, and toxicity [20]. Thus, considering the impact of a high BMI on drug metabolism and elimination is crucial in drug dosing.

Extensive studies on drug transporters and metabolizing enzymes and their transcriptional and posttranscriptional regulation mechanisms provide insights into variations in PK [28]. Unfortunately, data obtained from mice diet-induced obesity (DIO) are often inconsistent. *More and Slitt* reported hepatic but not renal transporter expression alteration in DIO mice. Induction of efflux transporter expression (i.e., *ABCC3* and *ABCC4*) and decreased by 50% of *ABCC1* mRNA and protein expression was noted in the liver [21]. On the other hand, *Cheng et al.* have shown alternation in mRNA and protein expression of a few multidrug resistance-associated proteins (*Mrps*), the efflux transporters in both livers and kidneys of obese mice [22].

Exploring all underlying factors that affect the PK and pharmacodynamics of xenobiotics in a diverse population is essential for improving therapeutic efficiency and minimizing adverse events. Despite numerous pathophysiological alterations associated with obesity described in the literature, the complete impact of these alterations on drug transporters has yet to be fully explored. This study evaluated the influence of overweight and obesity on renal and liver drug transporters’ gene expression in humans since data obtained from preclinical models often exhibit significant discrepancies when compared to clinical studies [23]. Our motivation was driven by the importance of drug transporters in influencing drug disposition and PK and the potential for their altered function in the presence of disease [24].

## Materials and methods

### Human tissue specimens

Forty-two necropsy specimens of liver and kidney were obtained from the National Disease Research Interchange (Philadelphia, PA, USA). The Rutgers Biomedical Health Sciences Institutional Review Board approved this study as exempt research (Pro2019001020). Any tissue with evidence of disease (i.e., malignancy, cirrhosis) was not accepted. Tissue specimens were snap-frozen immediately after collection, shipped on dry ice, and stored at − 80 °C until analysis. Included with each specimen was the donor’s age, gender, race, BMI (kg/m^2^), body weight (kg), medical history, surgical history, concomitant diseases, medication list, date and time of death; cause of death; date and time of autopsy (including post-mortem interval [PMI]). Donors of tissue specimens were divided into control and study groups based on their weight category. The control group included subjects of a healthy weight with a BMI of 18.5–25 kg/m^2^; the study group consisted of overweight and obese individuals (BMI ≥ 25 kg/m^2^). Before the RNA extraction assay, a single tissue block (from a single patient) was removed from − 80 °C, placed on a tray with liquid nitrogen, and crushed into pieces. Several small pieces were immediately collected into a weigh boat on dry ice. After weighing, the tissue sample was immediately put in a small tube on dry ice. The specimens were on dry ice the entire time and were never allowed to thaw entirely during this procedure.

### RNA extraction and cDNA synthesis

Total RNA was extracted from the liver and kidney samples using the AllPrep DNA/RNA/Protein Mini Kit (Qiagen, San Diego, CA, USA; product no. 80004) and purified using the RNeasy Mini Kit (Qiagen, San Diego, CA, USA; product no. 74104). Both procedures were performed according to the manufacturer’s protocols. Both tissues were homogenized (approximately 30–35 mg per sample) in a dedicated buffer using TissueLyser (Qiagen, San Diego, CA, USA). The quality and quantity of RNA were determined photometrically. The yield was determined in duplicate at an absorbance of 260 nm using a NanoDrop 2000 (Thermo Scientific, Waltham, MA, USA); all samples showed 260/280 ratios exceeding 1.8. Complementary DNA (cDNA) was synthesized from 0.5 µg of total RNA (in a reverse transcribed polymerase chain reaction (RT-PCR)) using a commercial RT2 First Strand Kit (Qiagen, San Diego, CA, USA; product no. 330401) according to the manufacturer’s recommendations. The cDNA concentration was diluted, and 10 ng total RNA input was used per individual qPCR reaction.

### Quantitative PCR

Real-time quantitative PCR was performed for the analysis of 84 drug transporter genes and 5 housekeeping genes using 96-well plates for Human Drug Transporters RT2 Profiler PCR Array System, a SYBR-Green-based method (Qiagen, San Diego, CA, USA; product no. PAHS-070Z) in QuantStudio 7 Pro Real-Time PCR instrument (Thermo Scientific, Waltham, MA, USA). The gene list of the Human Drug Transporters RT2 Profiler PCR Array System used in this study is available on Qiagen’s website (https://geneglobe.qiagen.com/us/product-groups/rt2-profiler-pcr-arrays). Primers for two additional housekeeping genes (*18S rRNA* and *PPIA*) were selected from ‘off the shelf’ (Qiagen, San Diego, CA, USA; product no. 330001). The relative gene expression levels were determined using the ΔC_t_ method. The cut-off C_t_ was set at 35 cycles for all analyses. The geometric means of housekeeping genes *RPLP0* and *HPRT1* for the kidney and *RPLP0* and *GAPDH* for the liver were used as an internal reference to normalize the mRNA expression results.

### Stability of gene expression

All samples (control and study group) were used to calculate the average C_t_ value. The stability of the seven housekeeping genes (*ACTB*, *B2M*, *RPLP0*, *HPRT1*, *GAPDH*, *18SrRNA*, and *PPIA*) was evaluated by algorithms geNorm, NormFinder, BestKeeper, and the comparative ΔC_t_ method. We compared and ranked the tested candidates using an online analysis tool, RefFinder [25]. Based on the rankings from each algorithm, RefFinder assigns an appropriate value to an individual gene and calculates the geometric mean of their values for the overall final ranking of the reference genes to determine their stability.

### Statistical analysis

Fold-change in gene expression between groups was estimated by dividing the respective mean [2^∧^(−ΔC_t_)] values [26]. Results were considered of potential biological relevance when the fold-change was ≥ 1.5 or ≤-1.5. In the first step, the obtained fold-change results were screened and values exceeding the cut-off were identified for each tissue (kidney – 16 genes, liver – 18 genes). Then, log-transformed [2^∧^(−ΔC_t_)] values were checked for normality using the Shapiro-Wilk test. Depending on the distribution, data were compared using a Student’s t-test or Mann-Whitney U-test; a p-value < 0.05 was considered statistically significant (no mathematical correction was made for multiple comparisons). For variables with confirmed normal distribution, an F-test was performed to check for equal variances. Variables with equal variances between groups were compared with a Student’s t-test; if variances were found unequal, Welch test was applied. Correlations between log-normalized [2^∧^(−ΔC_t_)] values and BMI were identified using the Spearman’s rank test (*rho* is the Spearman’s coefficient of rank correlation), and correlation was accepted at the significance level *p* = 0.05. Statistical analysis was performed using GraphPad Prism (GraphPad Prism 10, GraphPad Software, Inc., Boston, MA, USA) and MedCalc (version 22.030, MedCalc Software Ltd, Osten, Belgium).

## Results

### Studied population

Liver and kidney samples were collected from 32 to 28 subjects. Clinical characteristics of 42 subjects respectively (some subjects provided both organs) included in the study are presented in Table 1. The population was divided into two groups: the control group (lean subjects; 18.5 ≤ BMI < 25 kg/m^2^) and the study group (overweight/obese subjects; BMI ≥ 25 kg/m^2^).

**Table 1:** Clinical characteristics of the studied population. Human kidney and liver samples were collected post-mortem from 28 and 32 individuals (National Disease Research Interchange, Philadelphia, PA, USA), respectively. Patients were divided into the control group (lean subjects; 18.5 ≤ BMI < 25 kg/m^2^) and the study group (overweight/obese subjects; BMI ≥ 25 kg/m^2^)

Variables	Kidney samples			Liver samples		
	Control group (lean) *n* = 13	Study group (BMI ≥ 25) *n* = 15	statistics	Control group (lean) *n* = 15	Study group (BMI ≥ 25) *n* = 17	statistics
Age (yr)	63.8 ± 10.2	56.8 ± 9.9	NS	66 (62.5–71.8)	65 (48.8–68.2)	NS
Sex						
Female (%)	23.1	26.7	NS	40.0	41.2	NS
Male (%)	76.9	73.3	NS	60.0	58.8	NS
BMI (kg/m^2^)	23.2 (21.9–24.1)	31.6 (26.8–36.3)	U = 0.00 *p* < 0.0001	22.3 (21.1–24.0)	31.2 (27.7–37.3)	U = 0.00 *p* < 0.0001
Body weight (kg)	68.8 ± 3.8	96.4 ± 26.6	t_(d)_ = 3.696 *p* = 0.0011	64.6 ± 7.8	96.3 ± 26.9	t_(d)_ = 4.652 *p* = 0.0001
Post-mortem interval (h)	12.9 ± 4.4	13.7 ± 3.7	NS	13.6 ± 3.0	13.3 ± 3.2	NS
Race						
African-American (%)	15.4	13.3	NS	6.7	11.8	NS
Caucasian (%)	84.6	80.0	NS	93.3	82.3	NS
Unknown (%)	0.0	6.7	NS	0.0	5.9	NS
Concomitant diseases						
Asthma (%)	23.1	26.7	NS	36.4	23.5	NS
COPD (%)	33.3	20.0	NS	33.3	11.8	NS
Diabetes type 1 (%)	0	0	-	0	5.9	NS
Diabetes type 2 (%)	7.7	13.3	NS	20.0	11.8	NS
Hypertension (%)	69.2	100.0	*p* = 0.0349	53.3	100.0	*p* = 0.0019
Heart failure (%)	0	0	-	13.3	11.8	NS
Thyroid disorder (%)	0	6.7	NS	13.3	5.9	NS
Depression (%)	15.4	13.3	NS	0	23.5	NS
Anxiety (%)	7.7	6.7	NS	0	11.8	NS
Joint pain (%)	15.4	13.3	NS	26.7	17.6	NS
CAD (%)	15.4	20.0	NS	6.7	17.6	NS

CAD - Coronary Artery Disease; COPD - Chronic Obstructive Pulmonary Disease; NS – non-significant; t_(d)_ – Welch test statistic (Student’s t-test with the correction for the unequal variances). Continuous data are expressed as mean ± SD or median (interquartile range). Continuous data that do not follow normal distribution (e.g., BMI) were compared using the Mann-Whitney U test. Data with confirmed normal distribution (e.g., body weight) were compared with the Student’s t-test or Welch test, depending on the equality of variances (checked with the F-test). Categorical data are expressed as percentages and were compared using a two-tailed Fisher’s exact test

### mRNA expression in the kidney and liver

**Fig. 1:**
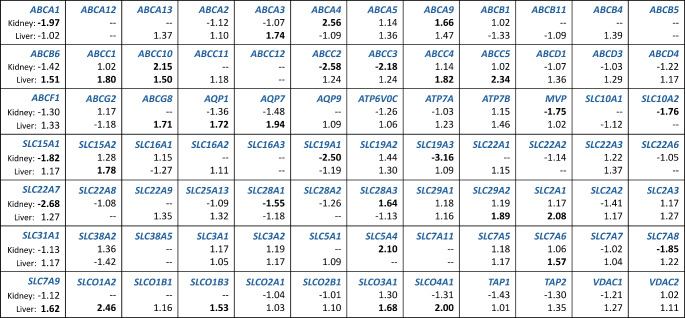
Drug transporter genes of which the mRNA expression in human kidneys and liver were evaluated, and the fold-change values. Human kidney and liver samples were collected post-mortem from 28 and 32 individuals (National Disease Research Interchange, Philadelphia, PA, USA), respectively. Patients were divided into the control group (lean subjects; 18.5 ≤ BMI < 25 kg/m^2^) and the study group (overweight/obese subjects; BMI ≥ 25 kg/m^2^). Each cell consists of the gene abbreviation marked blue (upper line), the fold-change value for the renal tissue (middle line), and the fold-change value for the liver tissue (lowest line). Fold-change in gene expression between groups was analyzed using qPCR and calculated by dividing the mean [2^∧^(−ΔC_t_)] value in the study group by the respective value for the control group. Results were considered of potential biological relevance if the fold-change was ≥ 1.5 or ≤-1.5; fold-change values above the cut-off are marked in bold

The mRNA expression of 84 genes encoding drug transporters in human kidneys and livers was evaluated (Fig. 1).

**Fig. 2:**
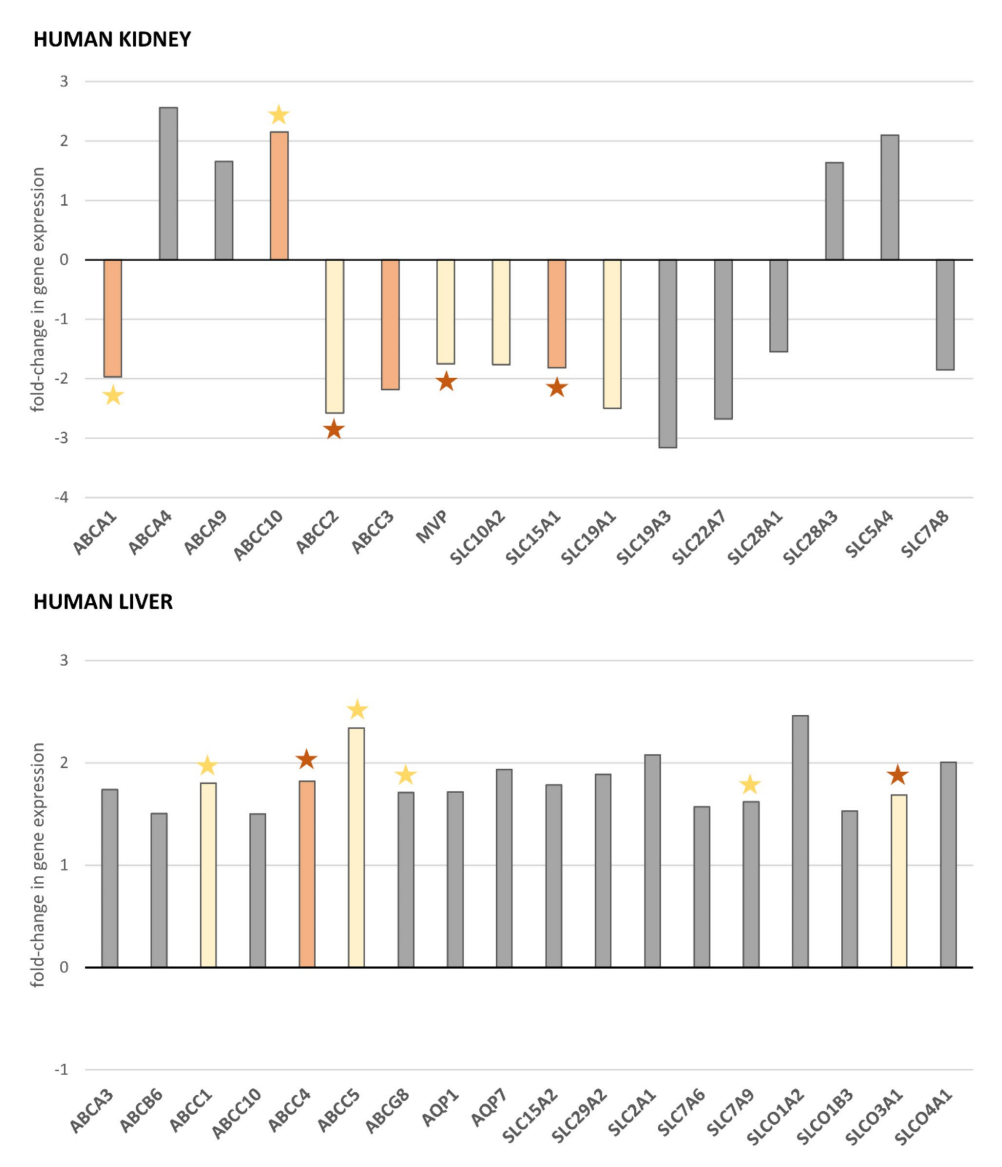
Drug transporter genes with potential biologically relevant differences in mRNA expression in the kidneys and liver between overweight/obese (BMI ≥ 25) and lean (18.5 ≤ BMI < 25) populations. Human kidney and liver samples were collected post-mortem from 28 and 32 individuals (National Disease Research Interchange, Philadelphia, PA, USA), respectively, which were divided into the control group (lean subjects; 18.5 ≤ BMI < 25 kg/m^2^) and the study group (overweight/obese subjects; BMI ≥ 25 kg/m^2^). Fold-change in gene expression between groups was analyzed using qPCR and calculated by dividing the respective mean [2^∧^(−ΔC_t_)] value for the study group by the value for the control group. Results were considered of potential biological relevance when the fold-change was ≥ 1.5 or ≤-1.5. Genes with a significant change (Student’s t-test or Mann-Whitney U-test; *p* < 0.05) in expression between groups are marked orange. Yellow color labels genes with a trend towards different expression (Student’s t-test or Mann-Whitney U-test; 0.05 ≤ *p* < 0.10). Grey color labels delineate results with no statistical significance (*p* > 0.10). Star indicates a correlation between the log-transformed [2^∧^(−ΔC_t_)] and BMI: orange star labels significant results (Spearman rank test, *p* < 0.05), yellow star indicates correlations close to significant (0.05 < *p* < 0.10). Statistics for the indicated differences and trends: KIDNEY (N_1_ = 13, N_2_ = 15) – *ABCC10* t_(d)_ = 2.366, *p* = 0.0277; *ABCC10* vs. BMI rho = 0.368, *p* = 0.0538; *SLC15A1* t_(d)_=-2.889, *p* = 0.0094; *SLC15A1* vs. BMI rho=-0.463, *p* = 0.0131; *ABCA1* U = 51.00, *p* = 0.0325; *ABCA1* vs. BMI rho=-0.318, *p* = 0.0991; *ABCC3* U = 53.00, *p* = 0.0413; *SLC10A2* t=-1.765, *p* = 0.0893; *ABCC2* U = 57.00, *p* = 0.0648; *MVP* U = 55.00, *p* = 0.0520; *MVP* vs. BMI rho=-0.475, *p* = 0.0106; *SLC19A1* U = 59.00, *p* = 0.0799; *SLC19A1* vs. BMI rho=-0.374, *p* = 0.0497; *SLCO3A1* U = 59.00, *p* = 0.0799; LIVER (N_1_ = 15, N_2_ = 17) – *ABCC4* U = 68.00, *p* = 0.0244; *ABCC4* vs. BMI rho = 0.383, *p* = 0.0303; *ABCC1* U = 78.00, *p* = 0.0637; *ABCC1* vs. BMI rho = 0.332, *p* = 0.0633; *ABCC5* U = 80.00, *p* = 0.0757; *ABCC5* vs. BMI rho = 0.317, *p* = 0.0770; *SLCO3A1* U = 82.00, *p* = 0.0894; *SLCO3A1* vs. BMI rho = 0.390, *p* = 0.0275; *ABCG8* vs. BMI rho = 0.306, *p* = 0.0888; *SLC7A9* rho = 0.307, *p* = 0.0872

Genes with very low expression (C_t_ >35) in more than 50% of control or/and study group subjects were excluded from further analysis. Fold-change in hepatic gene expression was calculated for 72 genes; relative change in renal gene expression was assessed for 64 genes. Screening for potential biological relevance was performed using fold-change cut-off 1.5 [27]. Fold-change analysis (Fig. 1) revealed 16 genes in the kidney and 18 genes in the liver, with values above the cut-off. Significant changes (Student’s t-test or Mann-Whitney U-test; *p* < 0.05) in mRNA expression were found in 4 genes in the kidney and 1 gene in the liver (Fig. 2).

**Fig. 3:**
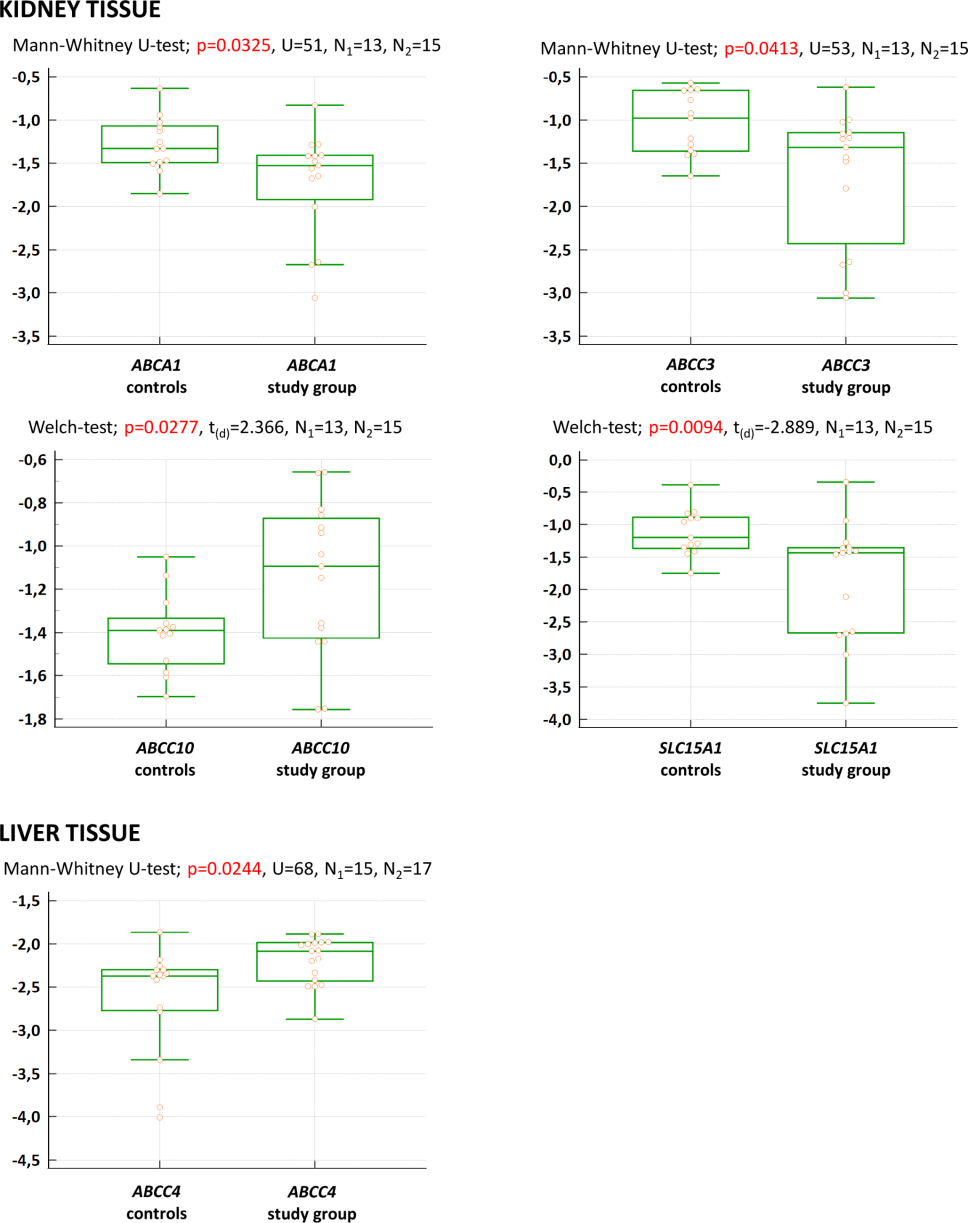
Differentially expressed genes of drug transporters in human kidney and liver. Human kidney and liver samples were collected post-mortem from 28 and 32 individuals (National Disease Research Interchange, Philadelphia, PA, USA), respectively, which were divided into the control group (lean subjects; 18.5 ≤ BMI < 25 kg/m^2^) and the study group (overweight/obese subjects; BMI ≥ 25 kg/m^2^). Relative expression was analyzed using qPCR and is shown as log-normalized [2^∧^(−ΔC_t_)] values: A box is drawn from the 1st to 3rd quartile, and a horizontal line is drawn at the median. The upper inner fence is the 3rd quartile + 1.5 × IQR; The lower inner fence is the 1st quartile – 1.5 IQR. The upper whisker is drawn at the highest value (observation, measurement) just below the upper inner fence. The lower whisker is drawn at the highest value (observation, measurement) just above the lower inner fence. The statistical test used for comparison with the respective results is shown in each single graph

In kidney tissue (Fig. 3), up-regulation was only observed for *ABCC10* (t_(d)_ = 2.366, *p* = 0.0277), while the other differentially expressed genes (DEGs) were down-regulated: *ABCA1* (U = 51, *p* = 0.0325), *ABCC3* (U = 53, *p* = 0.0413), and *SLC15A1* (t_(d)_=-2.889, *p* = 0.0094). In liver tissue (Fig. 3), the *ABCC4* gene was found up-regulated in overweight/obese subjects (U = 68, *p* = 0.0244).

Additionally, a trend towards lower mRNA expression in the kidney (Student’s t-test or Mann-Whitney U-test; 0.05 < *p* < 0.10) was observed for *ABCC2* (*p* = 0.0648; U = 57), *MVP* (*p* = 0.0520; U = 55), *SLC10A2* (*p* = 0.0893; t=-1.765) and *SLC19A1* (0.0799; U = 59). To further explore the relation of gene expression to obesity, the correlation of log-normalized [2^∧^(−ΔC_t_)] with BMI was verified with the Spearman test. Significant correlation was found for *SLC15A1* gene (*p* = 0.0131; rho=-0.463), *MVP* (*p* = 0.0106; rho=-0.475), *SLC19A1* (*p* = 0.0497; rho=-0.374). Correlation close to significant (0.05 < *p* < 0.10) was observed for *ABCA1* (*p* = 0.0991; rho=-0.318) and *ABCC10* (*p* = 0.0538; rho = 0.368).

**Fig. 4:**
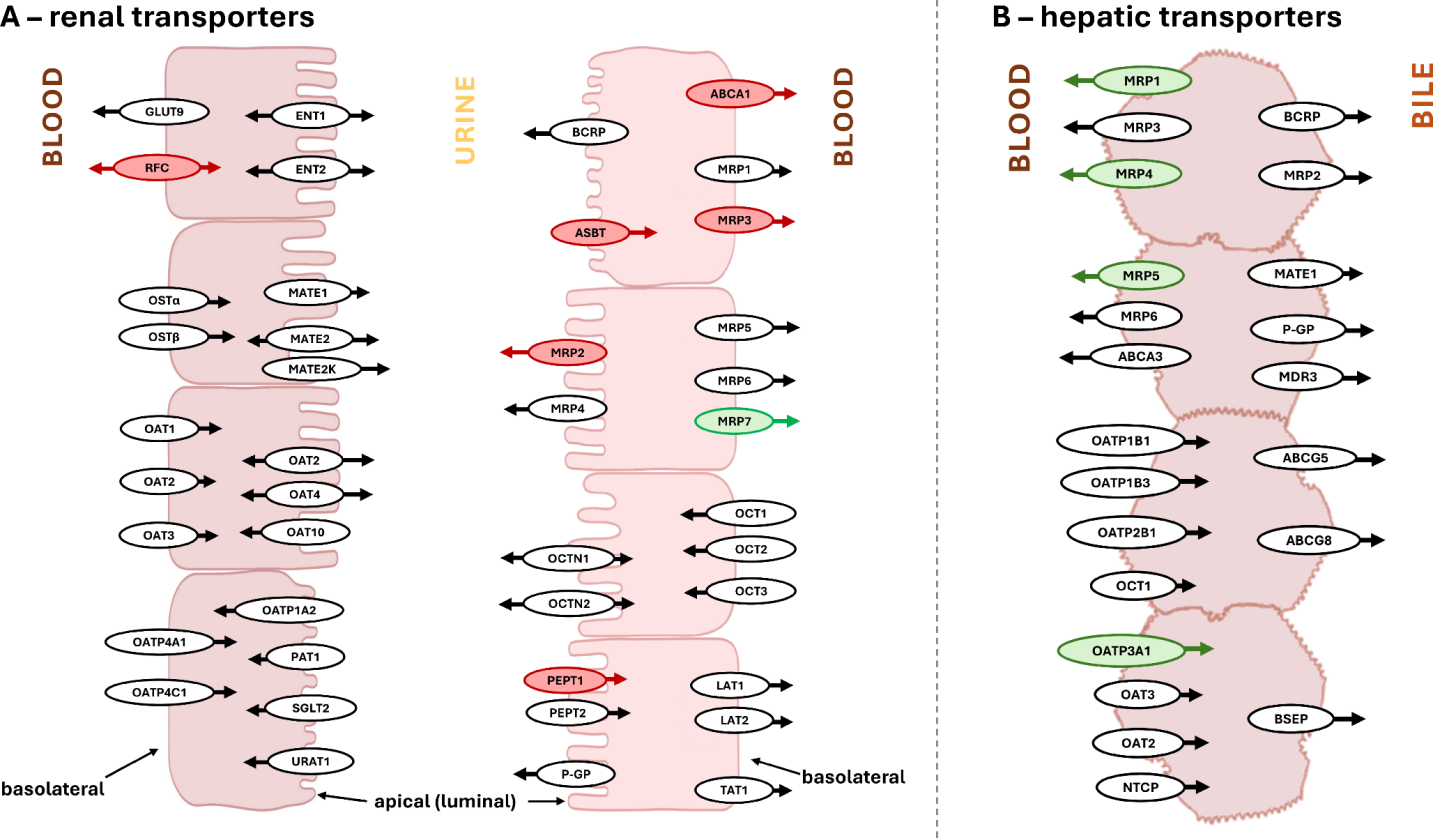
Localization of (**A**) renal and (**B**) hepatic transporters in humans [18, 28–33]. Red color labels transporters, which we found down-regulated (mRNA); green color labels up-regulated transporters. Symbol: ABCAs - subfamily A of ABC transporters, ASBT - apical sodium-dependent bile acid transporter, BCRP - breast cancer resistance protein, BSEP - bile salt export pump, ENTs - equilibrative nucleoside transporters, GLUT9 - facilitative glucose transporter 9, LATs - L-type amino-acid transporters, MATEs - multidrug and toxin extrusions, MRPs - multidrug resistance-associated proteins, NTCP - sodium taurocholate co-transporting polypeptide, OATs - organic anion transporters, OATPs - organic anion-transporting polypeptides, OCTNs - organic cation and carnitine transporters, OCTs - organic cation transporters, OSTs - organic solute transporters, PEPTs - peptide transporters, P-GP - P-glycoprotein (MDR1), RFC - reduced folate transporter, SGLT2 - sodium-glucose co-transporter 2, TAT1 - T-type amino acid transporter 1, URAT1 - urate transporter 1

A trend towards higher hepatic expression was observed for *ABCC1* (*p* = 0.0637; U = 78), *ABCC5* (*p* = 0.0757; U = 80), and *SLCO3A1* (*p* = 0.0894; U = 82). In the liver, a significant correlation with BMI was discovered for *ABCC4* (*p* = 0.0303; rho = 0.383) and *SLCO3A1* (*p* = 0.0275, rho = 0.390). Correlation close to significant was found for *ABCC1* (*p* = 0.0633; rho = 0.332), *ABCC5* (*p* = 0.0770; rho = 0.317), *ABCG8* (*p* = 0.0888; rho = 0.306), and *SLC7A9* (*p* = 0.0872; rho = 0.307). The main findings are summarized and reflected in Fig. 4.

## Discussion

Literature highlights the effects of obesity on drug PK by measuring the differences in PK parameters between lean and obese populations. Our work aimed to explore the underlying mechanisms, by screening for changes in the mRNA expression of 84 genes encoding human drug transporters in kidney and liver tissue. We found significant alterations in the expression of key ABC and SLC transporters in the kidney. The observations need to be further explored on the protein level, and may contribute to explaining differences in renal excretion of drugs and endogenous molecules between obese/overweight and lean individuals. Overexpression of ABC and SLC transporters in the liver was also observed which can help explain the reduction in hepatic clearance and potential drug resistance noted in the obese and overweight population. Overall, this study is the first to use human tissue samples from both obese/overweight and lean subjects to explore these differences in kidney and liver transporters.

### Renal excretion

Elimination of xenobiotics and endogenous molecules via urinary excretion involves three processes in the nephron, namely glomerular filtration, tubular secretion, and tubular reabsorption [5]. The influence of obesity on renal clearance is equivocal and requires considering the age of the patient. Initially, higher blood volume in obese subjects results in glomerular hyperfiltration and enhanced renal clearance. However, chronically elevated intra-glomerular pressure leads to glomerular lesions and, eventually, to chronic renal disease [5, 34]. Summary of studies showing altered drug PK in obesity concluded on possible augmented tubular secretion [34]. The shorter half-life of vancomycin in obese patients was related to the increased CL, and 80–90% of vancomycin elimination depends on renal excretion [8]. Tubular secretion and reabsorption involve transporters expressed on basolateral or apical membranes of tubule epithelia. These proteins are responsible for the unidirectional or bidirectional movement of both endo- and exogenous substrates and are classified into two superfamilies: ABC and SLC transporters [29, 30, 32]. The cellular site of expression, substrate affinity, and influx-efflux properties of the carrier determines how the transporter relates to the drug’s PK (Fig. 4A).

#### ATP-binding cassette (ABC) transporters

ABC transporters are expressed throughout the body, carry diverse substrates, and, therefore, are involved in various physiological and pathological processes. In renal tubule epithelial cells, ABC transporters export substrates out of the cells; when expressed on the apical membrane, they can be involved in tubular secretion and enhance renal clearance. When located on the basolateral membrane, the ABC transporters may add to reabsorption and diminish renal excretion [29, 30, 32]. Our study revealed significant differences in relative mRNA expression in the kidneys of several ABC transporters, which may explain drug PK differences between lean and obese individuals.

ABC transporters within subfamily C (ABCCs) include 9 proteins called Multidrug Resistance-associated Proteins (MRPs) due to their involvement in the efflux of various chemotherapeutics from cancer cells and the development of cancer multidrug resistance [35, 36]. Our study revealed that when compared to lean subjects renal expression of *ABCC3* (MRP3) was reduced and expression of *ABCC10* (MRP7) was increased in overweight/obese subjects; expression of *ABCC2* (MRP2) showed a significant negative correlation with BMI. *ABCC2* (MRP2) plays a substantial role in the elimination of drugs and toxic endogenous metabolites (e.g., antivirals, methotrexate, cisplatin, and angiotensin receptor blockers) due to its expression in the apical membrane of hepatocytes, small intestine, renal proximal tubules, and syncytiotrophoblast of the placenta [36, 37]. Our results suggest that within the overweight and obese population, there may be reduced renal secretion and increased risk of nephrotoxicity (impaired efflux from tubular epithelial cells) since *ABCC2* (MRP2) correlated significantly with BMI and showed a trend towards down-regulation (by 2.6-fold) in overweight and obese subjects when compared to lean individuals [31]. Additionally, this risk of nephrotoxicity is supported by our finding of a 2.2-fold down-regulation of *ABCC3* mRNA in the kidneys of obese/overweight subjects, which is also in support of findings from a murine model of obesity [38]. The diminished function of MRP3 could lead to a cumulation of xenobiotics in tubular epithelial cells and decrease renal reabsorption since MRP3 is localized in the basolateral cell membrane, which carries substrates (e.g., methotrexate, drug–glucuronides, drug-conjugates) out of the cell into the blood [36, 39]. Increased risk of nephrotoxicity in the obese and overweight population is also supported by our finding of overexpression (by 2.1-fold) of *ABCC10* (MRP7) mRNA, which MRP7 mediates the transport of substrates across the basolateral membrane from the cell into the blood. Overexpression of MRP7 could result in enhanced reabsorption of its substrates (i.e. anticancer therapies), reduced renal clearance, and potentially an increased risk of resistance to anticancer therapies [40].

Moreover, our study revealed *ABCA1*, which is important for efficient cholesterol efflux from the cell, to be expressed lower (by 2-fold) in overweight and obese individuals than in lean subjects. This result suggests that obese and overweight individuals could be at an increased risk of kidney disease since reduced *ABCA1* activity results in insufficient cholesterol excretion and accumulation of free cholesterol, which can damage cells and increase cell death [41–45]. 

#### Solute carrier (SLC) transporters

Similar to ABC transporters, SLC transporters mediate the uni- or bidirectional movement of endogenous compounds, drugs, and xenobiotics. Their primary function is the uptake of small molecules into cells, but they can also serve as efflux pumps (e.g., multidrug and toxin extrusions, MATEs) [32, 46, 47]. Their involvement in renal excretion of drugs depends on the site of expression – influx transporters are often (but not only) expressed at the basolateral membrane of renal epithelial cells. At the same time, efflux pumps are mainly localized at the luminal membranes of renal tubules (Fig. 4) [32]. Of importance our study showed that in obese/overweight individuals there is significant down-regulation (by 1.8-fold) of *SLC15A1* (encodes peptide transporter PEPT1) mRNA when compared to lean subjects; also, *SLC15A1* expression correlated significantly with BMI. PEPT1 is involved in the reabsorption of di- and tripeptides and is expressed apically in the small intestine, bile duct, and renal tubule epithelia [48, 49]. Given that PEPT1 mediates the uptake of peptidomimetic drugs, β-lactam antibiotics, and ACE inhibitors [48]; lower *SLC15A1* expression means decreased PEPT1 transporter expression leading to an augmented renal clearance due to diminished substrate renal reabsorption in obese/overweight individuals.

Noted differences in drug PK profiles between obese/overweight individuals and lean subjects could also be explained by changes in the expression of other SLC transporters. In our study, we observed that obese/overweight individuals had a trend for lower expression of *SLC10A2* (by 1.8-fold) when compared to lean subjects. *SLC10A2* encodes apical sodium-dependent bile acid transporter (ASBT), an apically localized uptake carrier expressed in the small intestine, that is responsible the for the reabsorption of bile acids and sterols in the proximal renal tubules [29, 50]. Lower expression of *SLC10A2* could mean that obese/overweight individuals experience an enhanced renal clearance of ASBT substrates since they are not reabsorbed. In addition, a trend for lower *SLC19A1* (by 2.5-fold), gene encoding reduced folate carrier protein (RFC), was observed in obese/overweight individuals. RFC in renal tubular cells is located in the basolateral membrane, which mediates the bidirectional transport of folates and clinically used antifolates [51, 52]. Given that obese/overweight individuals have lower expression of *SLC19A1*, RFC function could be diminished which in turn could lead to resistance of antifolates (i.e. chemotherapy) in this population [51, 52].

#### Other transporters

Further impairment in drug PK profiles in obese and overweight individuals, due to hinderance in renal clearance, could be explained by our finding of a significant negative correlation with BMI and a trend towards down-regulation of *MVP* (1.8-fold) in this population. *MVP* encodes a Major Vault Protein, a major component of vaults, and due to its expression in the cytoplasm is involved in cytoplasmic signal transmission pathways [53]. Seeing as vaults mediate multidrug resistance by transporting drugs away from their subcellular target, lower expression of MVP may mean a lower drug clearance from kidney cells and a greater risk of nephrotoxicity within the obese/overweight population.

### Hepatic uptake and biliary excretion

Hepatic clearance of drugs depends on the efficient transport across plasma membranes of hepatocytes, which is mediated by a large spectrum of transporters. Similarly to renal transporters, a particular hepatic carrier’s role in drug disposition is determined by substrate specificity, influx/efflux properties, and an expression site at the polarized cell membrane (Fig. 4B) [54]. The members of the ABC and SLC families predominantly mediate transmembrane transport in the liver [16].

#### ABC transporters

Changes in the expression of key ABC transporters within the liver, in conjunction with observed changes in the kidney can further the notion of what may be leading to differences in PK between lean and obese/overweight individuals. Within the liver *ABCC1* (MRP1), *ABCC4* (MRP4), and *ABCC5* (MRP5) are lowly expressed naturally [17, 55], however, in our study obese/overweight subjects showed significant overexpression of *ABCC4* (MRP4; 1.8 fold) and significant correlation with BMI; trends towards higher expression were found for *ABCC1* (MRP1; 1.8 fold) and ABCC5 (MRP5; 2.3-fold) [17, 55]. MRP1 transports a variety of endo- and exogenous substrates, which are structurally diverse (e.g., anthracyclines, Vinca alkaloids, methotrexate, rosuvastatin, quercetin) and protects tissues against damage from xenobiotics [33, 39]. Increased expression of MRP1 in the liver could result in enhanced efflux of its substrates back to the blood, leading to drug resistance (in case of efflux from target cells) or reduced hepatic clearance (in case of efflux from hepatocytes) in the obese/overweight population [33, 39]. Similar to MRP1, MRP4 is located basolaterally and involved in the efflux of drug metabolites or conjugates back into plasma (e.g., antiviral drugs, antibiotics, angiotensin receptor blockers, tamoxifen). Animal models of MRP4-knockout mice revealed that lack of MRP4 function was associated with adipocyte hypertrophy, increased adipose tissue mass, and increased blood glucose and leptin levels. MRP4-/- mice presented metabolic disease phenotype [56, 57]. *Donepudi et al.* concluded that *ABCC4* is a novel genetic factor involved in the development of metabolic diseases, including obesity and diabetes [56]. Interestingly, the cited results from the animal model suggest that obesity would be associated with lower MRP4 expression. However, our in-human study did not support this. Our findings corroborate with reports that hepatic expression of MRP4 was increased in non-alcoholic fatty liver disease which often accompanies obesity [58]. MRP5 in most cells is located basolaterally as it is for MRP1 and MRP4, but in the blood-brain barrier, it resides apically [59]. MRP5 carries cyclic nucleotides and folic acid, but can also transport anticancer nucleotide analogs [60] and thus may play a role in the efflux of these drugs back to plasma.

#### SLC transporters

Our study did not reveal significant differences in mRNA expression of genes encoding SLC transporters in the liver. However, a significant correlation between BMI and a trend towards overexpression (by 1.7-fold) of *SLCO3A1*, a gene encoding an organic anion transporter OATP3A1, was observed in the study group. In the liver OATP3A1 is relatively low; however, it elevates during cholestatic liver injury and serves as a protective bile acid efflux transporter [61]. Located at the basolateral membrane of hepatocytes, OATP3A1 facilitates the uptake of prostaglandins, thyroid hormones, vasopressin [62, 63], benzylpenicillin [63], and simvastatin [62]. Overexpression of OATP3A1 could result in enhanced uptake by hepatocytes and increased hepatic clearance of its substrates in the obese and overweight population.

### Summary and future directions

Our results show some significant changes in the expression of genes involved in human transporters, both renal and hepatic. The observed alterations may be reflected in differences in drug PK between lean and obese populations. However, these findings need further evaluation through the proteomic and functional study of these transporters in this patient population. Also, elucidating all underlying mechanisms and their clinical relevance is a long way to go. Translating the drug transporters research and knowledge into clinical pharmacology, patient care, and regulatory guidelines is as promising as challenging [64]. It was emphasized that one drug can be transported via various carriers, and identifying the most physiologically relevant pathway is difficult. Also, interpreting changes in transporters’ expression is challenging, as was proven for, e.g., methotrexate [32, 65], which is a substrate for several interplaying pathways. To our best knowledge, this is the first study in which a panel of 84 drug transporter genes was assessed in human liver and kidney samples. We aimed to initially explore the expression changes at the mRNA level to show the best future direction for research to explain PK alterations in patients with obesity. Our findings on altered mRNA expression in the kidney or liver would have to be further explored on the protein level, as it is well known that protein expression is regulated both on transcriptional and posttranscriptional levels. Available data on the correlation between mRNA and protein of drug transporters in human samples are equivocal. Semiquantitative PCR was confirmed useful for predicting protein expression of MRP1, MRP2, and MRP3, as the good agreement was reported for mRNA and protein levels in 23 cell lines of lung cancer [66]. A study on breast cancer cell lines showed a biostatistical trend (*p* = 0.069) between *ABCC3* expression and MRP3 protein level [67]. Expression of MRP4 protein followed the same pattern as *ABCC4* mRNA in prostate cancer cells [68] and renal cell carcinoma [69]. Reduced expression of *ABCA1* mRNA was paralleled by lower protein levels in human placental tissue [70], while in macrophages ABCA1 protein did not correlate with mRNA [71]. No significant correlation between ASBT protein and *SLC10A2* mRNA expression was found in human intestinal tissue [72]; on the contrary, the same expression pattern was observed in the human intestine for SLCO3A1 mRNA and OATP3A1 protein [73].

A considerable limitation of this study is that the expression analysis has been performed on post-mortem tissue, and therefore, the conclusions should be confronted with the possible natural RNA degradation. Several factors have been discussed to influence RNA integrity and RT-PCR results, but normalization can shrink this effect [74]. Therefore, as performing biopsies to collect healthy tissues from human volunteers could be ethically controversial and extremely difficult, we carefully analyzed patient characteristics for any confounders (Table 1) and used two reference genes to minimize the possible influence of RNA integrity on the expression results.

The patient cohort is both the strength and the limitation of our study. Clinical data show comorbidities, which may be a source of additional variability in results. However, both groups (controls and overweight/obese) are well matched in terms of age, sex, and clinical conditions, and what should be emphasized is that they accurately reflect the real-life population. Also, it has to be mentioned that the presented data needs further exploration. Observed changes in mRNA expression should be verified on a larger population and continued with the analysis of protein expression and function.

## Electronic supplementary material

Below is the link to the electronic supplementary material.

Supplementary Material 1

## Data Availability

The datasets generated during the current study are not publicly available due to ongoing application for research funding, which is related to the topic of this manuscript. The datasets are available from the corresponding author upon reasonable request.

## Abbreviations

ABC: ATP-Binding Cassette
ASBT: Apical Sodium-dependent Bile acid Transporter
BMI: Body Mass Index
CL: Clearance
cMOAT: Canalicular Multiple Organic Anion Transporter
DEG: Differentially Expressed Gene
DIO: Diet-Induced Obesity
LRP: Lung Resistance-related Protein
MATEs: Multidrug And Toxin Extrusions
MRP: Multidrug Resistance-associated Protein
PK: Pharmacokinetics
PMI: Post-Mortem Interval
RFC: Reduced Folate Carrier protein
RT-PCR: Reverse Transcribed Polymerase Chain Reaction
SLC: Solute Carrier
