# Supplementary material for: Obesity-related drug transporter expression alterations in human liver and kidneys
Source: Pharmacol Rep. 2024 Oct 16;76(6):1429–42. doi: 10.1007/s43440-024-00665-7 (PMC11582170; doi:10.1007/s43440-024-00665-7)
Supplement: Supplementary file 1 — Supplementary Material 1 [file 43440_2024_665_MOESM1_ESM.docx]

**Table S1**. The mean C_t_ values with standard deviation (SD) obtained in rt-PCR of human drug transporters in renal tissue. The results include the genes, for which the C_t_ values were above the cut-off (>35) in at least 50% of samples from the control and/or the study group.

| gene | Control group (n=13) | | Study group (n=15) | |
| --- | --- | --- | --- | --- |
|  | Mean | SD | Mean | SD |
| *ABCA1* | 31.47 | 1.26 | 34.14 | 3.37 |
| *ABCA2* | 31.85 | 1.43 | 34.49 | 4.30 |
| *ABCA3* | 31.24 | 1.34 | 32.69 | 2.66 |
| *ABCA4* | 36.68 | 3.23 | 36.95 | 4.04 |
| *ABCA5* | 29.30 | 0.89 | 30.37 | 2.19 |
| *ABCA9* | 31.93 | 1.20 | 32.94 | 2.38 |
| *ABCB1* | 28.95 | 1.03 | 30.15 | 2.61 |
| *ABCB6* | 30.19 | 1.56 | 31.96 | 2.01 |
| *ABCC1* | 30.31 | 0.92 | 31.43 | 1.87 |
| *ABCC10* | 31.87 | 1.33 | 32.23 | 1.18 |
| *ABCC2* | 29.87 | 1.51 | 32.96 | 3.94 |
| *ABCC3* | 30.67 | 1.96 | 33.89 | 4.25 |
| *ABCC4* | 29.76 | 1.35 | 30.78 | 2.29 |
| *ABCC5* | 27.91 | 1.09 | 29.17 | 1.88 |
| *ABCD1* | 33.16 | 2.32 | 35.21 | 3.75 |
| *ABCD3* | 28.90 | 1.17 | 30.13 | 2.46 |
| *ABCD4* | 28.25 | 0.78 | 29.72 | 1.67 |
| *ABCF1* | 29.86 | 1.31 | 31.66 | 3.13 |
| *ABCG2* | 34.47 | 3.28 | 35.50 | 3.95 |
| *AQP1* | 26.55 | 1.32 | 29.06 | 4.36 |
| *AQP7* | 30.73 | 0.96 | 32.98 | 3.43 |
| *ATP6V0C* | 26.47 | 1.58 | 27.94 | 2.41 |
| *ATP7A* | 32.43 | 1.54 | 35.20 | 4.31 |
| *ATP7B* | 33.96 | 2.88 | 35.36 | 3.60 |
| *MVP* | 30.94 | 2.07 | 33.51 | 3.99 |
| *SLC10A2* | 32.30 | 1.53 | 35.04 | 3.79 |
| *SLC15A1* | 30.93 | 1.82 | 34.57 | 4.32 |
| *SLC15A2* | 31.01 | 1.24 | 32.11 | 2.78 |
| *SLC16A1* | 30.92 | 1.55 | 32.86 | 3.42 |
| *SLC19A1* | 35.86 | 2.92 | 38.58 | 3.01 |
| *SLC19A2* | 29.72 | 1.59 | 30.30 | 1.60 |
| *SLC19A3* | 35.45 | 3.95 | 37.72 | 3.40 |
| *SLC22A2* | 28.63 | 1.20 | 29.80 | 2.34 |
| *SLC22A3* | 34.31 | 2.74 | 36.12 | 3.51 |
| *SLC22A6* | 27.49 | 1.38 | 29.28 | 2.71 |
| *SLC22A7* | 31.25 | 3.24 | 33.87 | 4.17 |
| *SLC22A8* | 28.88 | 1.91 | 30.44 | 2.76 |
| *SLC25A13* | 31.62 | 1.50 | 33.35 | 3.35 |
| *SLC28A1* | 28.71 | 1.66 | 30.44 | 2.25 |
| *SLC28A2* | 34.64 | 2.51 | 36.26 | 3.17 |
| *SLC28A3* | 36.83 | 3.07 | 38.44 | 2.72 |
| *SLC29A1* | 28.78 | 1.35 | 30.24 | 3.29 |
| *SLC29A2* | 31.45 | 1.12 | 32.81 | 3.33 |
| *SLC2A1* | 32.73 | 2.88 | 34.04 | 4.25 |
| *SLC2A2* | 29.04 | 1.67 | 30.74 | 1.97 |
| *SLC2A3* | 28.67 | 1.51 | 29.65 | 1.76 |
| *SLC31A1* | 32.64 | 2.50 | 35.10 | 4.38 |
| *SLC38A2* | 27.14 | 0.95 | 27.94 | 1.83 |
| *SLC3A1* | 26.97 | 1.62 | 28.10 | 2.55 |
| *SLC3A2* | 27.17 | 1.16 | 28.56 | 2.76 |
| *SLC5A4* | 36.24 | 3.13 | 37.96 | 3.05 |
| *SLC7A5* | 31.00 | 1.16 | 32.97 | 3.46 |
| *SLC7A6* | 32.04 | 2.58 | 33.29 | 3.27 |
| *SLC7A7* | 27.50 | 1.12 | 29.04 | 1.76 |
| *SLC7A8* | 26.97 | 1.18 | 28.58 | 1.90 |
| *SLC7A9* | 30.30 | 1.32 | 32.38 | 3.72 |
| *SLCO2A1* | 29.60 | 1.48 | 30.97 | 2.31 |
| *SLCO2B1* | 30.55 | 1.28 | 32.24 | 2.84 |
| *SLCO3A1* | 31.54 | 1.43 | 32.46 | 2.83 |
| *SLCO4A1* | 31.78 | 3.07 | 34.21 | 4.55 |
| *TAP1* | 30.69 | 1.55 | 32.87 | 3.56 |
| *TAP2* | 30.80 | 2.01 | 32.80 | 3.64 |
| *VDAC1* | 28.25 | 1.43 | 30.09 | 3.57 |
| *VDAC2* | 27.68 | 1.25 | 28.88 | 2.52 |

**Table S2**. The mean C_t_ values with standard deviation (SD) obtained in rt-PCR of human drug transporters in the liver tissue. The results include the genes, for which the C_t_ values were above the cut-off (>35) in at least 50% of samples from the control and/or the study group.

| gene | Control group (n=15) | | Study group (n=17) | |
| --- | --- | --- | --- | --- |
|  | Mean | SD | Mean | SD |
| *ABCA1* | 28.04 | 1.79 | 27.79 | 1.38 |
| *ABCA13* | 36.50 | 2.98 | 35.92 | 3.16 |
| *ABCA2* | 31.21 | 1.23 | 31.29 | 2.47 |
| *ABCA3* | 36.47 | 3.50 | 34.35 | 2.27 |
| *ABCA4* | 36.30 | 3.19 | 36.12 | 3.41 |
| *ABCA5* | 28.80 | 1.86 | 28.28 | 1.50 |
| *ABCA9* | 30.51 | 3.93 | 28.94 | 1.05 |
| *ABCB1* | 29.87 | 3.04 | 29.52 | 1.13 |
| *ABCB11* | 28.73 | 3.40 | 28.33 | 2.01 |
| *ABCB4* | 29.19 | 1.68 | 28.86 | 1.75 |
| *ABCB6* | 29.91 | 1.58 | 29.32 | 1.56 |
| *ABCC1* | 33.15 | 2.93 | 31.76 | 1.02 |
| *ABCC10* | 32.56 | 1.11 | 31.76 | 0.94 |
| *ABCC11* | 32.93 | 3.01 | 32.39 | 2.15 |
| *ABCC2* | 27.42 | 2.07 | 26.98 | 2.14 |
| *ABCC3* | 28.59 | 3.37 | 27.61 | 1.16 |
| *ABCC4* | 34.19 | 3.16 | 32.61 | 0.98 |
| *ABCC5* | 31.36 | 1.34 | 30.36 | 1.38 |
| *ABCD1* | 32.09 | 1.31 | 31.50 | 1.26 |
| *ABCD3* | 28.35 | 1.58 | 28.01 | 1.74 |
| *ABCD4* | 28.85 | 1.40 | 28.56 | 1.06 |
| *ABCF1* | 30.17 | 1.60 | 29.74 | 1.29 |
| *ABCG2* | 30.59 | 1.46 | 30.62 | 1.44 |
| *ABCG8* | 29.57 | 3.02 | 28.75 | 1.99 |
| *AQP1* | 30.37 | 1.85 | 29.69 | 1.54 |
| *AQP7* | 31.35 | 2.72 | 30.42 | 1.01 |
| *AQP9* | 28.40 | 1.78 | 28.38 | 2.04 |
| *ATP6V0C* | 28.28 | 1.53 | 28.09 | 1.20 |
| *ATP7A* | 34.50 | 2.99 | 34.06 | 2.93 |
| *ATP7B* | 31.87 | 2.45 | 31.72 | 3.27 |
| *MVP* | 33.20 | 3.74 | 32.39 | 2.42 |
| *SLC10A1* | 29.76 | 2.32 | 30.08 | 3.15 |
| *SLC15A1* | 30.03 | 1.72 | 30.04 | 1.47 |
| *SLC15A2* | 34.10 | 2.59 | 33.00 | 2.10 |
| *SLC16A1* | 28.92 | 1.75 | 29.13 | 1.87 |
| *SLC16A2* | 34.17 | 3.32 | 34.51 | 3.85 |
| *SLC19A1* | 33.66 | 2.69 | 33.75 | 3.21 |
| *SLC19A2* | 29.27 | 1.43 | 29.08 | 1.58 |
| *SLC19A3* | 32.72 | 3.25 | 31.87 | 1.38 |
| *SLC22A1* | 28.25 | 2.03 | 28.35 | 2.24 |
| *SLC22A3* | 30.06 | 1.37 | 29.78 | 1.86 |
| *SLC22A7* | 27.08 | 1.30 | 27.23 | 2.20 |
| *SLC22A9* | 28.72 | 1.60 | 28.54 | 1.85 |
| *SLC25A13* | 29.46 | 1.38 | 29.12 | 1.77 |
| *SLC28A1* | 27.75 | 3.52 | 27.63 | 1.62 |
| *SLC28A3* | 34.27 | 2.45 | 34.70 | 2.64 |
| *SLC29A1* | 28.55 | 1.20 | 28.44 | 1.17 |
| *SLC29A2* | 32.14 | 0.77 | 31.49 | 1.10 |
| *SLC2A1* | 35.80 | 3.22 | 35.85 | 3.73 |
| *SLC2A2* | 26.23 | 1.70 | 26.18 | 2.01 |
| *SLC2A3* | 28.31 | 2.43 | 28.12 | 1.57 |
| *SLC31A1* | 31.45 | 2.78 | 30.93 | 1.87 |
| *SLC38A2* | 25.52 | 2.11 | 25.71 | 1.54 |
| *SLC3A1* | 32.95 | 3.03 | 31.81 | 1.15 |
| *SLC3A2* | 30.34 | 3.20 | 29.45 | 1.44 |
| *SLC5A1* | 32.96 | 2.52 | 33.47 | 3.37 |
| *SLC7A5* | 30.11 | 1.38 | 29.28 | 1.09 |
| *SLC7A6* | 33.87 | 2.65 | 33.14 | 2.15 |
| *SLC7A7* | 29.21 | 1.28 | 29.14 | 1.28 |
| *SLC7A8* | 32.38 | 1.48 | 32.76 | 2.99 |
| *SLC7A9* | 32.57 | 2.35 | 32.01 | 2.42 |
| *SLCO1A2* | 31.17 | 2.62 | 30.37 | 2.90 |
| *SLCO1B1* | 27.35 | 1.69 | 26.96 | 1.38 |
| *SLCO1B3* | 29.49 | 2.96 | 28.78 | 3.67 |
| *SLCO2A1* | 34.84 | 3.43 | 35.17 | 3.36 |
| *SLCO2B1* | 26.35 | 1.23 | 26.36 | 1.70 |
| *SLCO3A1* | 32.65 | 2.46 | 31.75 | 1.02 |
| *SLCO4A1* | 34.96 | 3.87 | 34.52 | 3.86 |
| *TAP1* | 30.67 | 2.92 | 30.36 | 1.43 |
| *TAP2* | 31.27 | 1.67 | 30.99 | 1.59 |
| *VDAC1* | 28.85 | 1.78 | 28.35 | 1.39 |
| *VDAC2* | 28.98 | 1.47 | 28.77 | 1.19 |
